# Investigating linkage to care between hospitals and primary care clinics for people with TB in rural South Africa

**DOI:** 10.1371/journal.pone.0289830

**Published:** 2023-08-14

**Authors:** Tushar Garg, Bareng Aletta Sanny Nonyane, Mbali Mohlamonyane, Limakatso Lebina, Neil A. Martinson, David W. Dowdy, Colleen F. Hanrahan

**Affiliations:** 1 Department of Epidemiology, Johns Hopkins Bloomberg School of Public Health, Baltimore, Maryland, United States of America; 2 Department of International Health, Johns Hopkins Bloomberg School of Public Health, Baltimore, Maryland, United States of America; 3 Perinatal HIV Research Unit, University of the Witwatersrand, Johannesburg, South Africa; Stellenbosch University, SOUTH AFRICA

## Abstract

People with tuberculosis (TB) are often lost to follow-up during treatment transition to another facility. These losses may result in substantial morbidity and mortality but are rarely recorded. We conducted a record review on adults diagnosed with TB at 11 hospitals in Limpopo, South Africa, who were subsequently transferred to a local clinic to initiate or continue treatment. We then performed in-depth record reviews at the primary care clinic to which they were referred and called participants who could not be identified as starting treatment. Between August 2017 and April 2018, we reviewed records of 778 individuals diagnosed with TB in-hospital and later referred to local clinics for treatment. Of the 778, 88 (11%) did not link to care, and an additional 43 (5.5%) died. Compared to people without cough, those with cough had higher odds of linking to care (aOR = 2.01, 95% CI: 1.26–3.25, p = 0.005) and were also linked more quickly [adjusted Time Ratio (aTR) = 0.53, 95% CI:0.36–0.79, p<0.001], as were those diagnosed microbiologically (aOR = 1.86, 95% CI: 1.16–3.06, p = 0.012; aTR = 0.58, 95% CI: 0.34–0.98, p = 0.04). People diagnosed with TB in hospitals often disengage following referral to local clinics. Interventions to identify and re-engage people who do not present to local clinics within days of referral might close an important gap in the TB treatment cascade.

## Introduction

Tuberculosis (TB) is a global health crisis, with an estimated 10.6 million new cases and 1.6 million deaths in 2021 [1]. Losses throughout the cascade of diagnosis and treatment represent a major barrier to ending TB. While losses to follow-up during diagnosis and treatment are well described, transfer from diagnosis at hospitals to treatment at primary care clinics is an overlooked step in the TB care cascade. The World Health Organization recommends categories of TB treatment outcomes including cured, treatment completed, died, loss to care, treatment failure and transfer to another facility [2]. Relative to the other categories, those who transfer are poorly described and tracked. Further, there is no standard reporting on whether individuals who are transferred from one healthcare facility to another before or during treatment successfully link to local care, start on treatment and achieve favourable treatment outcomes.

In two rural health districts in Limpopo Province, South Africa, the majority (58%) of people receiving treatment for TB at primary health care clinics were transferred-in after TB diagnosis at other facilities, mainly hospitals [3]. People who transferred care in this fashion were more likely to be children, people living with HIV and TB-negative on their initial diagnostic test.

Understanding the magnitude and characteristics of people lost to care in the transfer process can help guide interventions to strengthen the TB care cascade, achieve successful treatment outcomes, and ultimately reduce transmission burden. We therefore sought to describe the linkage to TB care from hospital to primary care clinic, estimate the proportion of people referred who did not link to care, describe time to linkage-to-care, and identify individual predictors of unsuccessful linkage.

## Methods

### Study design

This retrospective cohort study was nested within Kharitode TB, a cluster-randomized comparative effectiveness trial comparing contact tracing and facility-based screening for active TB case finding in the rural districts of Vhembe and Waterberg, Limpopo province, South Africa [3]. This study was positioned in the six-month “washout” period during which facilities transitioned from contact tracing to facility-based screening (or vice versa).

We performed a record review of all patients diagnosed with TB at the 11 study hospitals in Vhembe and Waterberg districts that referred at least two patients to any of the 56 primary care clinics participating in Kharitode TB (S1 Table) for TB treatment. We then followed a cohort of people diagnosed with drug-sensitive TB at each study hospital from August 1, 2017 to April 30, 2018 via record review for linkage to care. Since we followed each patient to the clinic for linkage after enrolling during this eight-month duration, the follow-up time was variable for different participants. All participants were followed for a minimum of four months after TB diagnosis.

### Study population

The study population consisted of all individuals living in the catchment area of one of the 56 study clinics, who were diagnosed with active TB in a hospital and subsequently referred to receive treatment at a Kharitode TB study clinic. We included people of any age diagnosed with pulmonary or extrapulmonary TB using any diagnostic modality, including microbiologically-confirmed, radiologic, or clinical diagnosis. We excluded people with TB documented to be resistant to rifampin.

### Data collection

To identify the study population in corresponding hospitals, we used a combination of routine programmatic patient records, including sputum collection logs (at the referring hospital), electronic laboratory records (from the National Health Laboratory Service), and TB hospital and clinic treatment registers (both electronic and paper-based).

Study staff abstracted data from the above sources as well as patient medical files and transfer paperwork at hospitals and clinics. We used a combination of personal identifiers–including name, date of birth, and address–to link patients from hospital to the clinic to which they were referred. After verifying linkage, we abstracted data including age, sex, town or village of residence, date of diagnosis, mode of diagnosis (microbiologically confirmed or clinical), diagnostic test results including date of samples, referring clinic, site of TB (pulmonary or extrapulmonary), retreatment status, HIV status, ART usage at the time of diagnosis, TB symptoms, TB treatment regimen, date of TB treatment initiation, date of referral, and date of linkage to care at local clinic. (See case report form in S2 Table). The data were collected and accessed for research between January and July 2018. For each person, we accessed the source data in records only once.

We attempted to reach participants who could not be linked using clinical records by either calling the telephone number found in their records or by visiting their household if telephonic attempts were unsuccessful. We invited such people to participate in a short questionnaire to investigate whether they linked to care anywhere, and the reasons for linking to care at a different clinic or for not linking to care. The interview consisted of up to six questions (see follow-up case report form and participant interview script in S3 Table).

### Analysis

We defined time to linkage as the days elapsed from hospital discharge to initial presentation at the clinic. In our primary analysis, we excluded deaths and estimated time to linkage using cause-specific Accelerated Failure Time (AFT) models with robust standard errors to account for clustering at the hospital level [4]. The estimate Time ratio (TR) can be interpreted as percentage change in time to linkage per unit change in exposure variable. We applied multiple imputation with random-sample-observed values to impute time to linkage for patient records with an invalid or missing date of presentation at clinic. As a sensitivity analysis, we also estimated time to linkage to care using a competing-risk Accelerated Failure Time (AFT) model with Smooth Rank Regression using Gehan’s weight and Johnson and Strawderman’s sandwich variance estimates to account for the effect of death before linkage as a competing event [5, 6]. We used the R software package *aftgee*, using the aftsrr function weighted for censoring due to competing events and accounting for potential heterogenous errors due to hospital clustering [7]. We censored all observations at 90 days. We used a log-normal distribution for modelling time to linkage because this distribution showed the best fit and had the lowest Akaike Information Criterion (AIC) in comparison with other distributions in the intercept-only model. We further conducted an analysis on a binary outcome defined as linkage to care by 90 days. We used a logistic regression model with random effects for hospital clustering to assess individual characteristics that are associated with linkage. As an additional sensitivity analysis, we also performed all analyses on the complete-case dataset only.

We assessed multicollinearity among covariates by calculating the variance inflation factor. In all multivariable analyses, we assessed the association between the outcome and age; sex; HIV status; symptoms of cough, fever, weight loss, and night sweats; category and site of TB; length of hospital admission; and district. We defined statistical significance as two-sided p-value of <0.05. Analyses were performed in R version 4.1.2 (R Foundation for Statistical Computing, Vienna, Austria).

### Ethical considerations

The study was approved by the Human Research Ethics Committee at the University of the Witwatersrand in Johannesburg, South Africa and the Institutional Review Board at the Johns Hopkins Bloomberg School of Public Health, Baltimore, MD, USA. We received a waiver of informed consent for the record review. We obtained written and verbal informed consent for participants who were interviewed in-person at a household visit and over the phone, respectively.

## Results

**Fig 1:**
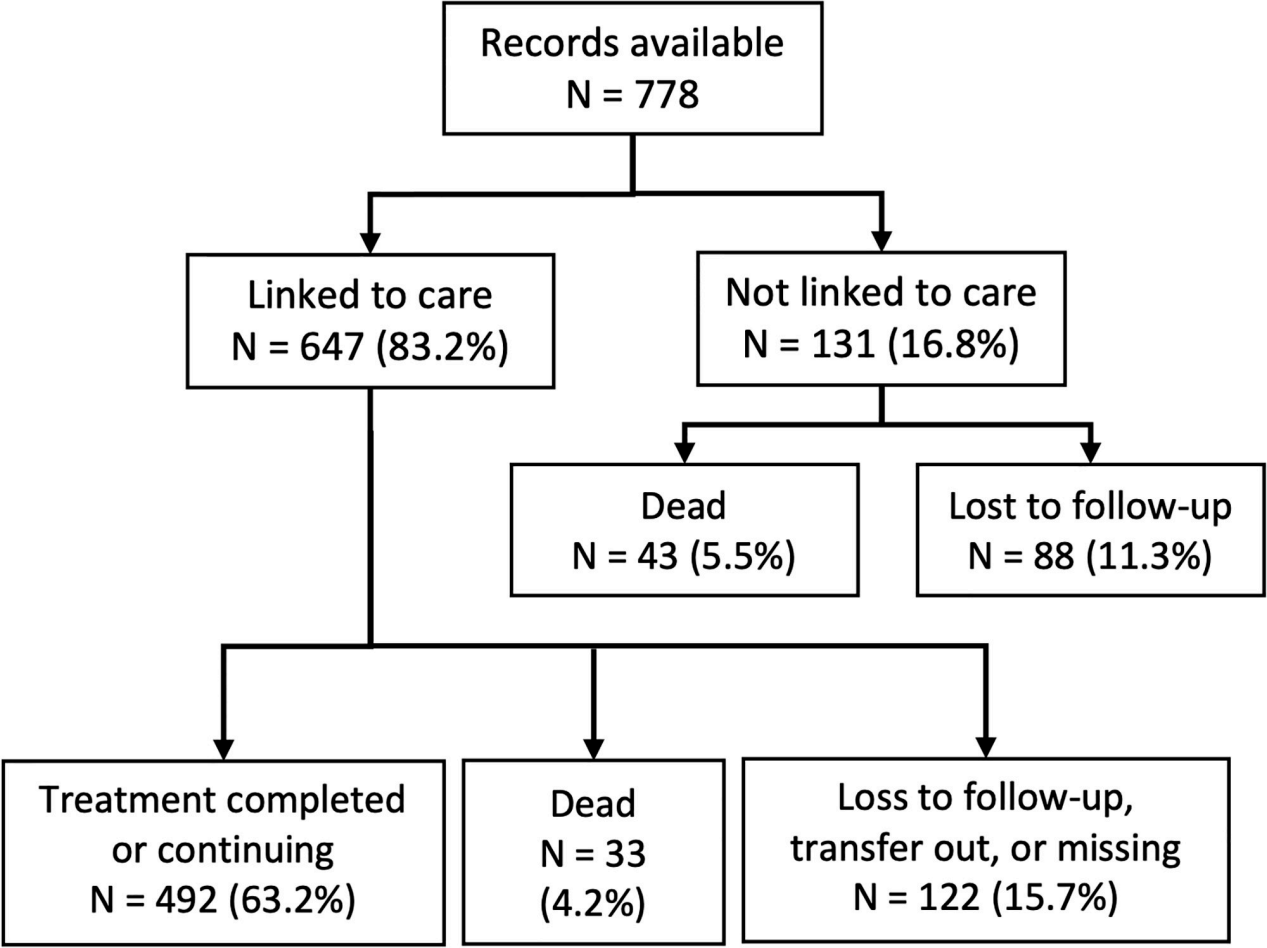
Patient flow diagram for study participants. The patient flow diagram represents the cohort of people diagnosed with drug-sensitive TB at each study hospital between August 1, 2017 and April 30, 2018 who were followed via record review for establishing linkage to care. All participants were followed for a minimum of four months after TB diagnosis. Since the follow-up time varied for patients, not all patients had completed treatment by the time of record review.

**Fig 2:**
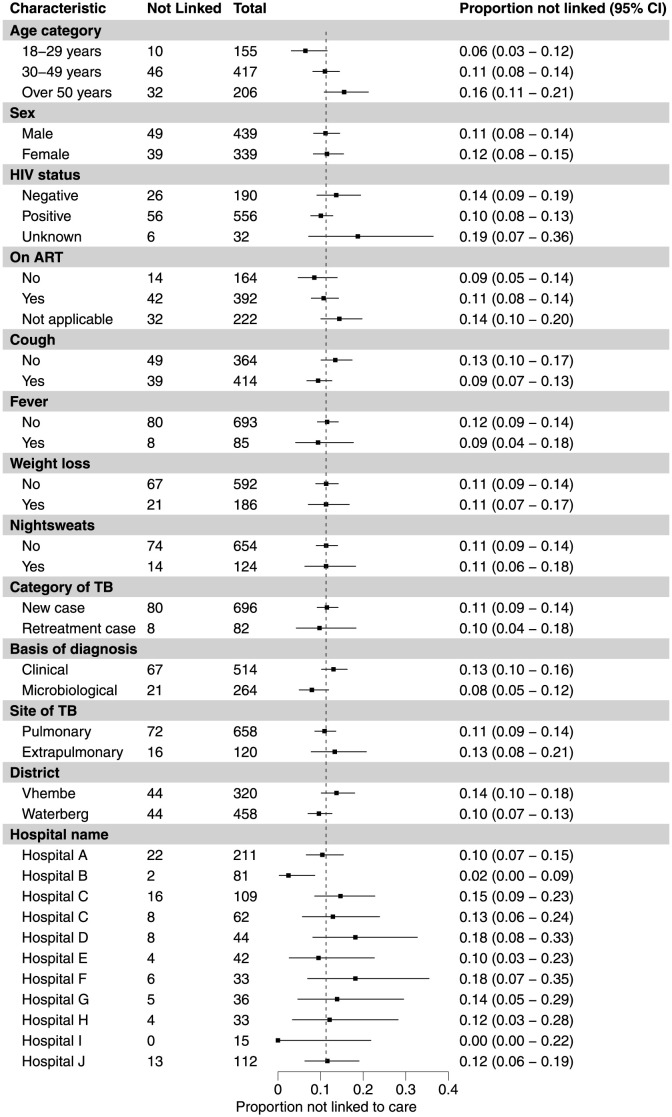
Proportion of patients diagnosed with TB in-hospital in rural South Africa but not linked to treatment at local clinics. This graph shows the proportion of patients who did not link to care, according to a-priori defined subgroups. Overall, 11.3% (88/778) people with TB did not link to care. Linkage to care was lower among patients who were older, did not report cough at baseline, and did not have microbiological confirmation of TB.

The study population included 778 patients who were referred from 11 hospitals in Vhembe and Waterberg districts to the 56 primary care clinics in the study area (Table 1). Of these, 88 patients (11%) did not continue TB treatment at the primary care clinic to which they were referred, an additional 43 (5.5%) died before linkage to care, and the remaining 647 (83%) linked to care (Figs 1 and 2). Among the 647 patients who successfully linked to care, 90 (14%) had a missing or invalid date of presentation. The date of death was missing for 19 (44%) of the 43 who died (S4 Table).

**Table 1:** Characteristics of the study population.

		District		
Characteristic	N(%) = 778	Vhembe, N = 320	Waterberg, N = 458	p-value
**Linkage to care status**				0.19
Died	43 (5.5%)	16 (5.0%)	27 (5.9%)	
Linked at clinic	647 (83%)	260 (81%)	387 (84%)	
Not linked to care	88 (11%)	44 (14%)	44 (9.6%)	
**Age category**				0.30
18–29 years	155 (20%)	72 (22%)	83 (18%)	
30–49 years	417 (54%)	164 (51%)	253 (55%)	
Over 50 years	206 (26%)	84 (26%)	122 (27%)	
**Sex**				0.37
Male	439 (56%)	174 (54%)	265 (58%)	
**HIV status**				<0.001
Negative	190 (24%)	107 (33%)	83 (18%)	
Positive	556 (71%)	205 (64%)	351 (77%)	
Unknown	32 (4.1%)	8 (2.5%)	24 (5.2%)	
**On ART**				<0.001
No	164 (21%)	26 (8.1%)	138 (30%)	
Yes	392 (50%)	179 (56%)	213 (47%)	
Not applicable	222 (29%)	115 (36%)	107 (23%)	
**Cough**	414 (53%)	193 (60%)	221 (48%)	0.001
**Fever**	85 (11%)	39 (12%)	46 (10%)	0.41
**Weight loss**	186 (24%)	84 (26%)	102 (22%)	0.23
**Night Sweats**	124 (16%)	62 (19%)	62 (14%)	0.037
**Category of TB**				0.32
New case	696 (89%)	291 (91%)	405 (88%)	
Retreatment case	82 (11%)	29 (9.1%)	53 (12%)	
**Basis of diagnosis**				<0.001
Microbiological	264 (34%)	147 (46%)	117 (26%)	
**Site of TB**				<0.001
Pulmonary	658 (85%)	242 (76%)	416 (91%)	
**Length of admission (days)** [Median (IQR)]	5 (0, 11)	8 (4, 15)	2 (0, 7)	<0.001

p-values were calculated using the Pearson’s Chi-squared test

**Fig 3:**
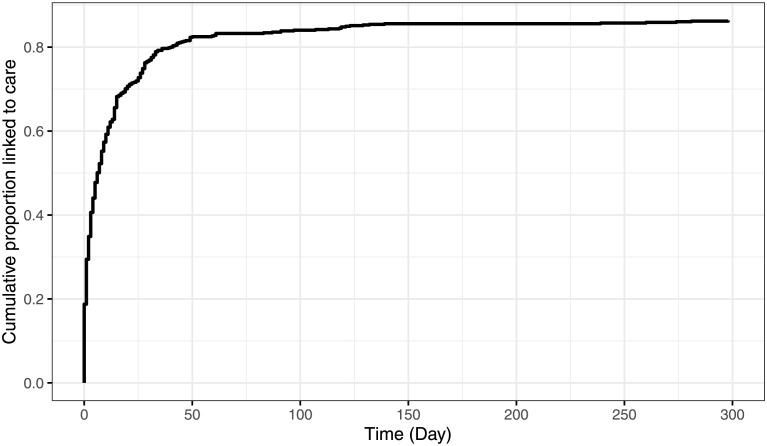
Time from hospital referral to presentation for initiation of treatment in rural South African clinics. The black line represents the proportion of people who were referred from hospitals to local clinics for TB treatment and had successfully linked to care, by the number of days post-discharge shown on the x-axis. Of the 647 who linked to care, the plot does not include 90 people with missing or invalid date of presentation. The median time to linkage for the 557 people who linked to care and had a valid date of presentation was 4 days (IQR: 1, 14).

The median time to linkage for the 557 (71.6%) people who linked to care and had a valid date of presentation was 4 days (IQR: 1, 14) (Fig 3). In the adjusted AFT model, the time to linkage was shorter by half for those reporting cough, compared to those without cough [adjusted Time Ratio (aTR) = 0.53, 95% CI:0.36–0.79, p<0.001]. Similarly, people whose TB was microbiologically confirmed linked to care faster than those diagnosed clinically (aTR = 0.58, 95% CI: 0.34–0.98, p = 0.04) (Table 2). Results were similar when modeling death as a competing risk (S5 Table). No measured characteristic was significantly associated with the risk of death among referred patients (S6 Table).

**Table 2:** Characteristics associated with time to linkage from hospital referral to local treatment initiation for TB in rural South Africa.

	Univariate			Multivariable		
Characteristic	TR^a^	95% CI	p-value	aTR^b^	95% CI	p-value
**Age category**						
18–29 years	Ref.			Ref.		
30–49 years	0.85	(0.42, 1.74)	0.66	0.94	(0.43, 2.05)	0.88
Over 50 years	1.24	(0.5, 3.1)	0.64	1.43	(0.59, 3.49)	0.43
**Sex**						
Female	1.23	(0.79, 1.91)	0.37	1.13	(0.74, 1.73)	0.56
**HIV status**						
Negative	Ref.			Ref.		
Positive	1.02	(0.6, 1.74)	0.94	0.97	(0.59, 1.61)	0.92
Unknown	0.48	(0.09, 2.57)	0.39	0.46	(0.11, 1.96)	0.29
**On ART**						
No	Ref.			—		
Yes	1.01	(0.49, 2.08)	0.98	—		
Not applicable	0.89	(0.4, 2.01)	0.78	—		
**Cough**	0.48	(0.31, 0.73)	<0.001	0.53	(0.36, 0.79)	<0.001
**Fever**	0.58	(0.22, 1.57)	0.29	0.75	(0.29, 1.93)	0.56
**Weight loss**	0.7	(0.34, 1.45)	0.34	1.06	(0.47, 2.41)	0.89
**Nightsweats**	0.61	(0.33, 1.11)	0.1	0.79	(0.4, 1.57)	0.51
**Category of TB**						
Retreatment case	1.64	(0.98, 2.73)	0.06	1.44	(0.81, 2.54)	0.21
**Basis of diagnosis**						
Microbiological	0.58	(0.33, 1.02)	0.06	0.58	(0.34, 0.98)	0.04
**Site of TB**						
Extrapulmonary	1.36	(0.69, 2.7)	0.37	1.14	(0.53, 2.48)	0.74
**Length of admission (days)**	1	(0.99, 1.02)	0.67	1	(0.99, 1.02)	0.71
**District**						
Waterberg	1.03	(0.51, 2.08)	0.94	0.86	(0.51, 1.46)	0.58

This analysis includes 647 people who linked to care.

^a^TR = Time Ratio

^b^aTR = adjusted Time Ratio

People who linked to care within 90 days were younger (adjusted odds ratio comparing ≥50 to <30 years old, aOR = 0.37, 95% CI: 0.18–0.71, p = 0.005) and had higher odds of self-reported cough (aOR = 2.01, 95% CI: 1.26–3.25, p = 0.005), microbiologically confirmed diagnosis (aOR = 1.86, 95% CI: 1.16–3.06, p = 0.012), and residence in Waterberg district (aOR = 1.61, 95% CI: 1.02–2.56, p = 0.041) (S7 Table). There was no evidence of statistically significant random variation by hospital. The sensitivity analysis results using complete-case data were consistent with the primary analysis (S8–S11 Tables).

## Discussion

In this study of 778 people referred out from rural South African hospitals for TB treatment, approximately one in six transferees either died or were lost to follow-up before presenting to the local clinic. People over 50 years old were less likely to link to care, whereas people reporting a cough or with microbiological confirmation were both more likely to link to care and did so faster (in about half the time as their counterparts without a cough or microbiologically confirmed TB). These findings illustrate the challenges faced by older individuals in linking to TB care at local clinics and suggest potentially lower motivation to link to treatment among people with fewer symptoms or no laboratory confirmation of their disease. They also suggest potential target populations for interventions to strengthen the care cascade at the time of referral from hospital to clinic.

Prior estimates of linkage to care at primary care clinics after discharge from hospitals vary widely. Studies in Kwazulu Natal, Western Cape, and Gauteng reported loss to care during linkage of 29%, 36%, and 50%, respectively [8–10]. In comparison, through detailed record review and interview of participants, we were able to confirm linkage to care (or death) in 89% of our rural study population. Our results of better linkage following microbiological diagnosis are consistent with similar findings from the Western Cape [9]. Regarding higher linkage among people with symptomatic cough and microbiological confirmation, these individuals are also more likely to experience more severe symptoms in general, likely increasing the probability of self-directed attempts to seek care at primary care clinics even when they are unaware of, or unable to complete, the initial referral [11].

Qualitative evidence from Western Cape and Cape Town highlights shortcomings in person-centered care, particularly in the discharge and linkage process [11–13]. People with TB often do not receive adequate resources (e.g., financial support, detailed instructions, contact information) to successfully complete referrals; this is particularly true for people from marginalized communities or with low health literacy. Weak coordination between staff at referring hospitals and local clinics adds to this challenge [8]. Interventions that adopt a person-centered care approach while addressing health system challenges can improve continuity of care [14]. For example, creating an exclusive care and coordination center for people with TB at a tertiary care hospital in Gauteng improved linkage to treatment from 50% to 93% [15]. Similarly, a comprehensive intervention in Western Cape that prepared patients at discharge through education, counselling and coordination with primary care clinics improved linkage from 40% to 92% [16].

We used patient records maintained by the health facilities and the national TB program to identify eligible participants and extract information on demographics and TB diagnosis. While valuable in understanding real-world performance, routine data are often not maintained to a high standard of quality. For example, date information was missing or incorrect in 17% of our records. In these cases, we imputed times to linkage based on observed values–but these imputed results could lead to bias in our results. Further, our accounting of deaths is likely an underestimate because some people may have died after linkage to care or interview, and it is likely that some of those for whom we were unable to ascertain linkage or vital status had died. Lastly, socioeconomic characteristics may influence the pathway to care, but the absence of socioeconomic data in routine patient records precluded analysis of this association.

In conclusion, referral following TB diagnosis in hospitals to local clinics for treatment is an important–and often overlooked–step in the TB care cascade that is associated with high losses to follow-up. Gaps in successful linkage were particularly notable for patients who were older, did not report a cough at the time of referral, or did not have microbiological confirmation of TB. These findings can aid in the development of interventions to strengthen the TB care cascade, particularly for these patients who may be at increased risk of loss to follow-up during referral.

## Supporting information

S1 Table Study hospitals. In the study population, 52.2% had transferred-in to care from another facility, of whom 84% came from a hospital, 14% a clinic or private doctor, and data was missing for the remaining 1%. The 56 primary care clinics were purposively chosen based on volume of TB cases in the year prior to study, completeness of TB record keeping, and willingness to participate.
(DOCX)

S2 Table Case report form. (DOCX)

S3 Table Case report form for follow-up and participant interview script. (DOCX)

S4 Table Information on missingness of date data for the study population. Invalid date of presentation indicates that date of presentation at clinic was earlier than the date of discharge from hospital.
(DOCX)

S5 Table Characteristics associated with time to linkage from hospital referral to local treatment initiation for TB in rural South Africa. This analysis used competing-risk accelerated failure time model after imputing median time to linkage for missing values. Death was treated as an event with competing risk. Time to linkage to care was censored at 90 days.
(DOCX)

S6 Table Characteristics associated with death of people with TB referred from hospital to local clinic for treatment initiation in rural South Africa. This analysis considers deaths as all people who died during the study duration irrespective of time awaiting linkage to care or linkage to care status. People who died (n = 76) includes 43 and 33 people who died before and after linking to care, respectively.
(DOCX)

S7 Table Characteristics associated with linkage to care from hospital referral to local treatment initiation for TB in rural South Africa. Linkage to care is defined as people initiating treatment at a local clinic within 90 days of referral from the hospital. Where date of presentation or death was missing or invalid, time to linkage or death has been imputed by median of valid values for respective variable. This analysis does not include people who died within 90 days (n = 40).
(DOCX)

S8 Table Characteristics associated with linkage to care from hospital referral to local treatment initiation for TB in rural South Africa. This is based on complete case dataset including 557 who linked to care and 91 who did not link to care. This analysis does not include people who died within 90 days (n = 21). Linkage to care is defined as people initiating treatment at a local clinic within 90 days of referral from the hospital.
(DOCX)

S9 Table Characteristics associated with death of people with TB referred from hospital to local clinic for treatment initiation in rural South Africa. This is based on complete case dataset (n = 669). This analysis considers deaths as all people who died during the study duration irrespective of time awaiting linkage to care or linkage to care status. Factors associated with death in the complete case analysis. People who died (n = 55) includes 24 and 31 people who died before and after linking to care, respectively.
(DOCX)

S10 Table Characteristics associated with time to linkage from hospital referral to local treatment initiation for TB in rural South Africa. This is based on complete case dataset (n = 669).
(DOCX)

S11 Table Characteristics associated with time to linkage from hospital referral to local treatment initiation for TB in rural South Africa. This is based on complete case dataset. (n = 669). This analysis used competing-risk accelerated failure time model after imputing median time to linkage for missing values. Death was treated as an event with competing risk. Time to linkage to care was censored at 90 days.
(DOCX)

S1 Checklist STROBE statement—Checklist of items that should be included in reports of observational studies. (DOCX)

S1 Data (CSV)
