# Supplementary material for: Investigating linkage to care between hospitals and primary care clinics for people with TB in rural South Africa
Source: PLoS One. 2023 Aug 14;18(8):e0289830. doi: 10.1371/journal.pone.0289830 (PMC10424851; doi:10.1371/journal.pone.0289830)
Supplement: S1 Table — In the study population, 52.2% had transferred-in to care from another facility, of whom 84% came from a hospital, 14% a clinic or private doctor, and data was missing for the remaining 1%. The 56 primary care clinics were purposively chosen based on volume of TB cases in the year prior to study, completeness of TB record keeping, and willingness to participate. (DOCX) [file pone.0289830.s001.docx]

# Supporting information

## S1 Table. Details of study hospitals and clinics

| **Hospital name** | **N** |
| --- | --- |
| Hospital A | 211 (27%) |
| Hospital B | 81 (10%) |
| Hospital C | 109 (14%) |
| Hospital D | 62 (8.0%) |
| Hospital E | 44 (5.7%) |
| Hospital F | 42 (5.4%) |
| Hospital G | 33 (4.2%) |
| Hospital H | 36 (4.6%) |
| Hospital I | 33 (4.2%) |
| Hospital J | 15 (1.9%) |
| Hospital K | 112 (14%) |

In the study population, 52.2% had transferred-in to care from another facility, of whom 84% came from a hospital, 14% a clinic or private doctor, and data was missing for the remaining 1%. The 56 primary care clinics were purposively chosen based on volume of TB cases in the year prior to study, completeness of TB record keeping, and willingness to participate.
