# Supplementary material for: Investigating linkage to care between hospitals and primary care clinics for people with TB in rural South Africa
Source: PLoS One. 2023 Aug 14;18(8):e0289830. doi: 10.1371/journal.pone.0289830 (PMC10424851; doi:10.1371/journal.pone.0289830)
Supplement: S2 Table — (DOCX) [file pone.0289830.s002.docx]

# Supporting information

## S2 Table. Case Report Form

Patient ID: ___ ___ -____ ____ ____

**At Hospital**

Hospital name: _________________

Sex: □ Male □ Female

DOB: dd/mmm/yyyy

Date of presentation at hospital: dd/mmm/yyyy

Referring facility: _________________

Date of referral: dd/mmm/yyyy

Diagnostic test results:

|  | Date of sample collection | Type of sample | Test | Test Result | Result date |
| --- | --- | --- | --- | --- | --- |
| Test 1 |  |  |  |  |  |
| Test 2 |  |  |  |  |  |
| Test 3 |  |  |  |  |  |
| Test 4 |  |  |  |  |  |
| Test 5 |  |  |  |  |  |

Radiologic results:

|  | Date of Xray | Radiologist notes | Suggestive of TB (Y/N) |
| --- | --- | --- | --- |
| Xray 1 |  |  |  |
| Xray 2 |  |  |  |
| Xray 3 |  |  |  |

Date of TB diagnosis: dd/mmm/yyyy

Basis of TB diagnosis : □ Microbiologically confirmed □ Radiologic □ Clinical

Site of TB: □ Pulmonary □Extrapulmonary (Site:_______________)

Retreatment status: □ New TB □Retreatment (Date of prior episode: mmm/yyyy

HIV status: □ Negative □Positive □ Unknown

Date of HIV diagnosis: dd/mmm/yyyy

ART use: □No □Yes

ART start date: dd/mmm/yyyy

Most recent CD4 count: ___________ cells/ml Date: dd/mmm/yyyy

Most recent viral load: ___________ copies/ml Date: dd/mmm/yyyy

TB symptoms

| Symptoms | Duration |
| --- | --- |
| □ Cough | ­­­­­______ days/weeks/months |
| □ Fever | ______ days/weeks/months |
| □ Weight loss | ______ days/weeks/months |
| □ Night sweats | ______ days/weeks/months |
| □ Other symptom: _____________ | ______ days/weeks/months |
| □ Other symptom: _____________ | ______ days/weeks/months |
| □ Other symptom: _____________ | ______ days/weeks/months |

TB treatment start date: dd/mmm/yyyy

TB regimen: ____________________

Date of referral: dd/mmm/yyyy

Facility referred to: __________________________

**At Referral Clinic**

Date of TB treatment initiation: dd/mmm/yyyy

TB regimen: ____________________

Additional diagnostic test results:

|  | Date of sample collection | Type of sample | Test | Test Result | Result date |
| --- | --- | --- | --- | --- | --- |
| Test 1 |  |  |  |  |  |
| Test 2 |  |  |  |  |  |
| Test 3 |  |  |  |  |  |
| Test 4 |  |  |  |  |  |
| Test 5 |  |  |  |  |  |

Radiologic results:

|  | Date of Xray | Radiologist notes | Suggestive of TB (Y/N) |
| --- | --- | --- | --- |
| Xray 1 |  |  |  |
| Xray 2 |  |  |  |
| Xray 3 |  |  |  |

TB treatment outcome: □ Completed □ Cured □ Failed □ Died □Lost to Care □ Transfer out ( Where:________)

Treatment outcome date: dd/mmm/yyyy
