# Supplementary material for: Investigating linkage to care between hospitals and primary care clinics for people with TB in rural South Africa
Source: PLoS One. 2023 Aug 14;18(8):e0289830. doi: 10.1371/journal.pone.0289830 (PMC10424851; doi:10.1371/journal.pone.0289830)
Supplement: S3 Table — (DOCX) [file pone.0289830.s003.docx]

# Supporting information

## S3 Table. Case Report Form for follow-up and participant interview script

| Type of follow-up: | - Call | | - Visit | | |
| --- | --- | --- | --- | --- | --- |
| Time of call: | | | ___ ___ : ___ ___ | | |
| Name of study person making the call: | | | ____________________________________ | | |
| Follow-up outcome: | | | | | |
| - Successful | | - No answer | | - On voicemail | - New number |
| - Other: ____________________________ | | | | | |

Person spoken to: □ Participant □ Household member/relative/friend

*Confirm Identity of participant*

*Name: Similar to our records:*

| - Yes | - No |
| --- | --- |

*Date of Birth: Similar to our records*

| - Yes | - No |
| --- | --- |

*In attempting to contact this individual, I learned that they died on dd-mm-yyy [END INTERVIEW]*

Thank you for agreeing to participate. According to the records at _____________ Hospital, on <date> you were referred to ___________ Clinic in order to continue TB treatment. According to the records at _______ Clinic, you did not get TB treatment in that clinic.

1. Where, if anywhere, did you go to continue treatment for this episode of TB?

a. I went to a different clinic than the one you mentioned

b. I went to a private doctor [End interview here]

c. I went to a traditional healer or faith healer [END INTERVIEW HERE]

b. I did not go anywhere to continue treatment [Skip to Q 5)

2. What clinic did you go to for TB treatment, instead of _______ Clinic?

______________

3. What is the town/village that is closest to this clinic?

______________

4. When did you start TB treatment at this clinic?

dd-mmm-yyyy

5. Why did you go to a different clinic for TB treatment than the one the hospital referred you to?

a. The clinic was more convenient to work or home

b. The clinic has friendlier staff or gives better care

c. The clinic was recommended by a friend or family member

d. The clinic is outside my community, so I would not run into anyone I know.

e. Other, specify:_______________________________________________

6. How long does it take you (one-way) to travel to the clinic where you are receiving treatment?

______ minutes

7. What would have made it easier for you to continue treatment after you were discharged from the hospital?

______________________________________________________________________________

*[END INTERVIEW HERE]*

8. Why did you end your TB treatment after leaving the hospital?

a. I felt better and did not need care

b. I was too busy or could not make time to get to the clinic

c. I was travelling and could not get to any clinic

d. I was worried about being seen going to the clinic by people I know

e. I don’t like going to a clinic for medical treatment

f. I was too sick to go to the clinic

g. Other, specify:_______________________________________________

9. Do you have any of the following symptoms:

a. Cough (y/n)

b. Fever (y/n)

c. Night sweats (y/n)

d. Weight loss (y/n)

10. What would have made it easier for you to continue treatment after you were discharged from the hospital?

___________________________________________________________________________
