# Supplementary material for: Investigating linkage to care between hospitals and primary care clinics for people with TB in rural South Africa
Source: PLoS One. 2023 Aug 14;18(8):e0289830. doi: 10.1371/journal.pone.0289830 (PMC10424851; doi:10.1371/journal.pone.0289830)
Supplement: S4 Table — Invalid date of presentation indicates that date of presentation at clinic was earlier than the date of discharge from hospital. (DOCX) [file pone.0289830.s004.docx]

# Supporting information

## S4 Table. Information on missingness of date data for the study population

| **Linkage to care** | **Date information** | **n** |
| --- | --- | --- |
| Linked at clinic | Valid date of presentation at clinic | 557 |
|  | Invalid date of presentation at clinic | 55 |
|  | Missing data of presentation at clinic | 35 |
| Not linked to care |  | 88 |
| Died | Valid date of death | 24 |
|  | Missing date of death | 19 |
| Total |  | 778 |

Invalid date of presentation indicates that date of presentation at clinic was earlier than the date of discharge from hospital.
