# Supplementary material for: Investigating linkage to care between hospitals and primary care clinics for people with TB in rural South Africa
Source: PLoS One. 2023 Aug 14;18(8):e0289830. doi: 10.1371/journal.pone.0289830 (PMC10424851; doi:10.1371/journal.pone.0289830)
Supplement: S5 Table — This analysis used competing-risk accelerated failure time model after imputing median time to linkage for missing values. Death was treated as an event with competing risk. Time to linkage to care was censored at 90 days. (DOCX) [file pone.0289830.s005.docx]

# Supporting information

## S5 Table. Characteristics associated with time to linkage from hospital referral to local treatment initiation for TB in rural South Africa

|  | Univariate | | | Multivariable | | |
| --- | --- | --- | --- | --- | --- | --- |
| Characteristic | TR^a^ | 95% CI | p-value | aTR^b^ | 95% CI | p-value |
| **Age category** |  |  |  |  |  |  |
| 18-29 years | Ref. |  |  | Ref. |  |  |
| 30-49 years | 1.01 | (0.57, 1.76) | 0.98 | 1.22 | (0.43, 3.47) | 0.71 |
| Over 50 years | 1.54 | (0.58, 4.05) | 0.38 | 2.22 | (0.61, 8.05) | 0.23 |
| **Sex** |  |  |  |  |  |  |
| Female | 1.09 | (0.84, 1.42) | 0.5 | 1.04 | (0.56, 1.94) | 0.89 |
| **HIV status** |  |  |  |  |  |  |
| Negative | Ref. |  |  | Ref. |  |  |
| Positive | 1.01 | (0.77, 1.32) | 0.97 | 0.99 | (0.43, 2.29) | 0.98 |
| Unknown | 0.99 | (0.34, 2.92) | 0.98 | 0.93 | (0.2, 4.26) | 0.92 |
| **On ART** |  |  |  |  |  |  |
| No | Ref. |  |  | — |  |  |
| Yes | 1.14 | (0.84, 1.54) | 0.4 | — |  |  |
| Not Applicable | 1.08 | (0.7, 1.68) | 0.72 | — |  |  |
| **Cough** | 0.53 | (0.41, 0.69) | <0.001 | 0.46 | (0.33, 0.64) | <0.001 |
| **Fever** | 0.6 | (0.3, 1.23) | 0.17 | 0.66 | (0.36, 1.23) | 0.19 |
| **Weight loss** | 0.88 | (0.7, 1.1) | 0.25 | 1.39 | (0.82, 2.35) | 0.22 |
| **Nightsweats** | 0.69 | (0.45, 1.05) | 0.08 | 0.74 | (0.2, 2.77) | 0.66 |
| **Category of TB** |  |  |  |  |  |  |
| Retreatment case | 1.05 | (0.61, 1.81) | 0.85 | 0.83 | (0.35, 1.99) | 0.68 |
| **Basis of diagnosis** |  |  |  |  |  |  |
| Microbiological | 0.59 | (0.38, 0.92) | 0.02 | 0.43 | (0.22, 0.82) | 0.01 |
| **Site of TB** |  |  |  |  |  |  |
| Extrapulmonary | 1.26 | (0.93, 1.7) | 0.13 | 1 | (0.56, 1.79) | 1 |
| **Length of admission (days)** | 1.01 | (0.97, 1.04) | 0.62 | 1.01 | (0.97, 1.05) | 0.68 |
| **District** |  |  |  |  |  |  |
| Waterberg | 0.81 | (0.5, 1.31) | 0.39 | 0.59 | (0.3, 1.14) | 0.12 |

This analysis used competing-risk accelerated failure time model after imputing median time to linkage for missing values. Death was treated as an event with competing risk. Time to linkage to care was censored at 90 days.

^a^TR = Time Ratio

^b^aTR = adjusted Time Ratio
