# Supplementary material for: Investigating linkage to care between hospitals and primary care clinics for people with TB in rural South Africa
Source: PLoS One. 2023 Aug 14;18(8):e0289830. doi: 10.1371/journal.pone.0289830 (PMC10424851; doi:10.1371/journal.pone.0289830)
Supplement: S6 Table — This analysis considers deaths as all people who died during the study duration irrespective of time awaiting linkage to care or linkage to care status. People who died (n = 76) includes 43 and 33 people who died before and after linking to care, respectively. (DOCX) [file pone.0289830.s006.docx]

# Supporting information

## S6 Table. Characteristics associated with death of people with TB referred from hospital to local clinic for treatment initiation in rural South Africa

|  | Univariate | | | | Multivariable | | |
| --- | --- | --- | --- | --- | --- | --- | --- |
| Characteristic | n/N | OR^a^ | 95% CI | p-value | aOR^b^ | 95% CI | p-value |
| **Age category** |  |  |  |  |  |  |  |
| 18-29 years | 14/155 | Ref. |  |  | Ref. |  |  |
| 30-49 years | 36/417 | 0.95 | 0.51, 1.87 | 0.88 | 0.89 | 0.47, 1.77 | 0.7 |
| Over 50 years | 26/206 | 1.45 | 0.74, 2.96 | 0.29 | 1.48 | 0.75, 3.05 | 0.3 |
| **Sex** |  |  |  |  |  |  |  |
| Female | 28/339 | 0.73 | 0.44, 1.19 | 0.21 | 0.71 | 0.42, 1.16 | 0.2 |
| **HIV status** |  |  |  |  |  |  |  |
| Negative | 15/190 | Ref. |  |  | Ref. |  |  |
| Positive | 58/556 | 1.36 | 0.77, 2.54 | 0.31 | 1.61 | 0.87, 3.12 | 0.13 |
| Unknown | 3/32 | 1.21 | 0.27, 3.95 | 0.78 | 1.11 | 0.24, 3.73 | 0.9 |
| **On ART** |  |  |  |  |  |  |  |
| No | 16/164 | Ref. |  |  | — |  |  |
| Yes | 42/392 | 1.11 | 0.62, 2.09 | 0.74 | — |  |  |
| Not applicable | 18/222 | 0.82 | 0.40, 1.67 | 0.57 | — |  |  |
| **Cough** | 36/414 | 0.77 | 0.48, 1.24 | 0.28 | 0.79 | 0.46, 1.34 | 0.4 |
| **Fever** | 6/85 | 0.68 | 0.26, 1.49 | 0.38 | 0.74 | 0.26, 1.80 | 0.5 |
| **Weight loss** | 18/186 | 0.99 | 0.55, 1.69 | 0.96 | 1.34 | 0.68, 2.55 | 0.4 |
| **Nightsweats** | 9/124 | 0.69 | 0.31, 1.35 | 0.31 | 0.65 | 0.27, 1.45 | 0.3 |
| **Category of TB** |  |  |  |  |  |  |  |
| Retreatment case | 6/82 | 0.71 | 0.27, 1.56 | 0.43 | 0.60 | 0.22, 1.35 | 0.2 |
| **Basis of diagnosis** |  |  |  |  |  |  |  |
| Microbiological | 22/264 | 0.77 | 0.45, 1.28 | 0.34 | 0.78 | 0.44, 1.32 | 0.4 |
| **Site of TB** |  |  |  |  |  |  |  |
| Extrapulmonary | 11/120 | 0.92 | 0.45, 1.73 | 0.81 | 0.86 | 0.41, 1.70 | 0.7 |
| **Length of admission (days)** | 5/5 | 1.00 | 0.98, 1.01 | 0.56 | 1.00 | 0.98, 1.01 | 0.6 |
| **District** |  |  |  |  |  |  |  |
| Waterberg | 47/458 | 1.15 | 0.71, 1.88 | 0.58 | 0.94 | 0.54, 1.62 | 0.8 |

This analysis considers deaths as all people who died during the study duration irrespective of time awaiting linkage to care or linkage to care status. People who died (n = 76) includes 43 and 33 people who died before and after linking to care, respectively.

^a^OR = Odds Ratio

^b^aOR = adjusted Odds Ratio
