# Supplementary material for: Investigating linkage to care between hospitals and primary care clinics for people with TB in rural South Africa
Source: PLoS One. 2023 Aug 14;18(8):e0289830. doi: 10.1371/journal.pone.0289830 (PMC10424851; doi:10.1371/journal.pone.0289830)
Supplement: S8 Table — This is based on complete case dataset including 557 who linked to care and 91 who did not link to care. This analysis does not include people who died within 90 days (n = 21). Linkage to care is defined as people initiating treatment at a local clinic within 90 days of referral from the hospital. (DOCX) [file pone.0289830.s008.docx]

# Supporting information

## S8 Table. Characteristics associated with linkage to care from hospital referral to local treatment initiation for TB in rural South Africa

|  |  | Univariate |  |  | Multivariable |  |
| --- | --- | --- | --- | --- | --- | --- |
| Characteristic | OR^a^ | 95% CI | p-value | aOR^b^ | 95% CI | p-value |
| **Age category** |  |  |  |  |  |  |
| 18-29 years | Ref. |  |  | Ref. |  |  |
| 30-49 years | 0.65 | 0.33, 1.18 | 0.17 | 0.52 | 0.26, 0.98 | 0.059 |
| Over 50 years | 0.43 | 0.22, 0.83 | 0.014 | 0.37 | 0.18, 0.72 | 0.005 |
| **Sex** |  |  |  |  |  |  |
| Female | 0.78 | 0.52, 1.18 | 0.24 | 0.71 | 0.46, 1.10 | 0.11 |
| **HIV status** |  |  |  |  |  |  |
| Negative | Ref. |  |  | Ref. |  |  |
| Positive | 1.29 | 0.80, 2.04 | 0.29 | 1.37 | 0.80, 2.30 | 0.2 |
| Unknown | 0.79 | 0.31, 2.32 | 0.65 | 0.81 | 0.30, 2.48 | 0.7 |
| **On ART** |  |  |  |  |  |  |
| No | Ref. |  |  | — |  |  |
| Yes | 0.76 | 0.41, 1.35 | 0.37 | — |  |  |
| Not applicable | 0.62 | 0.32, 1.13 | 0.13 | — |  |  |
| **Cough** | 1.77 | 1.17, 2.70 | 0.007 | 1.88 | 1.16, 3.09 | 0.016 |
| **Fever** | 1.29 | 0.66, 2.75 | 0.48 | 1.15 | 0.54, 2.65 | 0.7 |
| **Weight loss** | 1.00 | 0.63, 1.65 | 0.99 | 0.75 | 0.42, 1.39 | 0.4 |
| **Nightsweats** | 1.14 | 0.67, 2.07 | 0.64 | 1.02 | 0.52, 2.09 | >0.9 |
| **Category of TB** |  |  |  |  |  |  |
| Retreatment case | 1.76 | 0.87, 4.08 | 0.15 | 1.79 | 0.86, 4.21 | 0.15 |
| **Basis of diagnosis** |  |  |  |  |  |  |
| Microbiological | 1.48 | 0.94, 2.37 | 0.10 | 1.87 | 1.15, 3.12 | 0.014 |
| **Site of TB** |  |  |  |  |  |  |
| Extrapulmonary | 0.70 | 0.42, 1.21 | 0.19 | 1.02 | 0.57, 1.88 | >0.9 |
| **Length of admission (days)** | 0.98 | 0.97, 1.00 | 0.012 | 0.99 | 0.97, 1.00 | 0.036 |
| **District** |  |  |  |  |  |  |
| Waterberg | 1.46 | 0.96, 2.21 | 0.073 | 1.54 | 0.96, 2.47 | 0.075 |

This is based on complete case dataset including 557 who linked to care and 91 who did not link to care. This analysis does not include people who died within 90 days (n = 21). Linkage to care is defined as people initiating treatment at a local clinic within 90 days of referral from the hospital.

^a^OR = Odds Ratio, ^b^aOR = adjusted Odds Ratio
