# Supplementary material for: Investigating linkage to care between hospitals and primary care clinics for people with TB in rural South Africa
Source: PLoS One. 2023 Aug 14;18(8):e0289830. doi: 10.1371/journal.pone.0289830 (PMC10424851; doi:10.1371/journal.pone.0289830)
Supplement: S9 Table — This is based on complete case dataset (n = 669). This analysis considers deaths as all people who died during the study duration irrespective of time awaiting linkage to care or linkage to care status. Factors associated with death in the complete case analysis. People who died (n = 55) includes 24 and 31 people who died before and after linking to care, respectively. (DOCX) [file pone.0289830.s009.docx]

# Supporting information

## S9 Table. Characteristics associated with death of people with TB referred from hospital to local clinic for treatment initiation in rural South Africa

|  | Univariate | | | | Multivariable | | |
| --- | --- | --- | --- | --- | --- | --- | --- |
| Characteristic | n/N | OR^a^ | 95% CI | p-value | aOR^b^ | 95% CI | p-value |
| **Age category** |  |  |  |  |  |  |  |
| 18-29 years | 9/131 | Ref. |  |  | Ref. |  |  |
| 30-49 years | 26/358 | 1.06 | 0.50, 2.46 | 0.88 | 0.94 | 0.43, 2.21 | 0.9 |
| Over 50 years | 20/180 | 1.69 | 0.77, 4.03 | 0.21 | 1.73 | 0.77, 4.17 | 0.2 |
| **Sex** |  |  |  |  |  |  |  |
| Female | 20/291 | 0.72 | 0.40, 1.27 | 0.27 | 0.72 | 0.39, 1.27 | 0.3 |
| **HIV status** |  |  |  |  |  |  |  |
| Negative | 10/166 | Ref. |  |  | Ref. |  |  |
| Positive | 42/475 | 1.51 | 0.77, 3.26 | 0.26 | 1.81 | 0.87, 4.07 | 0.12 |
| Unknown | 3/28 | 1.87 | 0.40, 6.63 | 0.37 | 1.71 | 0.36, 6.26 | 0.4 |
| **On ART** |  |  |  |  |  |  |  |
| No | 12/135 | Ref. |  |  | — |  |  |
| Yes | 30/340 | 0.99 | 0.50, 2.07 | 0.98 | — |  |  |
| Not applicable | 13/194 | 0.74 | 0.32, 1.69 | 0.46 | — |  |  |
| **Cough** | 28/354 | 0.92 | 0.53, 1.60 | 0.76 | 0.97 | 0.52, 1.80 | >0.9 |
| **Fever** | 5/73 | 0.80 | 0.27, 1.91 | 0.65 | 0.85 | 0.26, 2.31 | 0.7 |
| **Weight loss** | 12/158 | 0.89 | 0.44, 1.69 | 0.74 | 1.02 | 0.45, 2.20 | >0.9 |
| **Nightsweats** | 8/113 | 0.83 | 0.35, 1.71 | 0.63 | 0.78 | 0.29, 1.91 | 0.6 |
| **Category of TB** |  |  |  |  |  |  |  |
| Retreatment case | 5/75 | 0.78 | 0.26, 1.84 | 0.60 | 0.68 | 0.23, 1.63 | 0.4 |
| **Basis of diagnosis** |  |  |  |  |  |  |  |
| Microbiological | 16/222 | 0.81 | 0.43, 1.46 | 0.50 | 0.80 | 0.41, 1.48 | 0.5 |
| **Site of TB** |  |  |  |  |  |  |  |
| Extrapulmonary | 5/99 | 0.55 | 0.19, 1.30 | 0.22 | 0.52 | 0.17, 1.29 | 0.2 |
| **Length of admission (days)** | 5/4 | 1.00 | 0.98, 1.02 | 0.86 | 1.00 | 0.98, 1.02 | 0.7 |
| **District** |  |  |  |  |  |  |  |
| Waterberg | 34/396 | 1.13 | 0.64, 2.02 | 0.68 | 0.89 | 0.47, 1.70 | 0.7 |

This is based on complete case dataset (n=669). This analysis considers deaths as all people who died during the study duration irrespective of time awaiting linkage to care or linkage to care status. Factors associated with death in the complete case analysis. People who died (n = 55) includes 24 and 31 people who died before and after linking to care, respectively.

^a^OR = Odds Ratio, ^b^aOR = adjusted Odds Ratio
