# Supplementary material for: Investigating linkage to care between hospitals and primary care clinics for people with TB in rural South Africa
Source: PLoS One. 2023 Aug 14;18(8):e0289830. doi: 10.1371/journal.pone.0289830 (PMC10424851; doi:10.1371/journal.pone.0289830)
Supplement: S10 Table — This is based on complete case dataset (n = 669). (DOCX) [file pone.0289830.s010.docx]

# Supporting information

## S10 Table. Characteristics associated with time to linkage from hospital referral to local treatment initiation for TB in rural South Africa

|  | Univariate | | |  | Multivariable |  |
| --- | --- | --- | --- | --- | --- | --- |
| Characteristic | TR^a^ | 95% CI | p-value | aTR^b^ | 95% CI | p-value |
| **Age category** |  |  |  |  |  |  |
| 18-29 years | Ref. |  |  | Ref. |  |  |
| 30-49 years | 1.15 | 0.61, 2.18 | 0.67 | 1.35 | 0.71, 2.58 | 0.4 |
| Over 50 years | 1.88 | 0.84, 4.21 | 0.12 | 2.20 | 1.03, 4.71 | 0.042 |
| **Sex** |  |  |  |  |  |  |
| Female | 1.26 | 0.92, 1.74 | 0.15 | 1.25 | 0.94, 1.65 | 0.12 |
| **HIV status** |  |  |  |  |  |  |
| Negative | Ref. |  |  | Ref. |  |  |
| Positive | 0.92 | 0.65, 1.30 | 0.63 | 0.89 | 0.62, 1.26 | 0.5 |
| Unknown | 0.95 | 0.26, 3.53 | 0.94 | 0.90 | 0.36, 2.24 | 0.8 |
| **On ART** |  |  |  |  |  |  |
| No | Ref. |  |  | — |  |  |
| Yes | 1.18 | 0.60, 2.29 | 0.63 | — |  |  |
| Not applicable | 1.22 | 0.71, 2.09 | 0.48 | — |  |  |
| **Cough** | 0.44 | 0.35, 0.55 | <0.001 | 0.47 | 0.36, 0.62 | <0.001 |
| **Fever** | 0.57 | 0.27, 1.18 | 0.13 | 0.70 | 0.31, 1.59 | 0.4 |
| **Weight loss** | 0.82 | 0.43, 1.56 | 0.54 | 1.29 | 0.60, 2.75 | 0.5 |
| **Nightsweats** | 0.67 | 0.43, 1.05 | 0.079 | 0.86 | 0.46, 1.59 | 0.6 |
| **Category of TB** |  |  |  |  |  |  |
| Retreatment case | 1.09 | 0.65, 1.82 | 0.76 | 1.00 | 0.57, 1.75 | >0.9 |
| **Basis of diagnosis** |  |  |  |  |  |  |
| Microbiological | 0.49 | 0.32, 0.74 | <0.001 | 0.41 | 0.26, 0.65 | <0.001 |
| **Site of TB** |  |  |  |  |  |  |
| Extrapulmonary | 1.54 | 0.97, 2.43 | 0.065 | 1.05 | 0.62, 1.80 | 0.9 |
| **Length of admission (days)** | 1.02 | 1.01, 1.02 | <0.001 | 1.02 | 1.01, 1.03 | 0.001 |
| **District** |  |  |  |  |  |  |
| Waterberg | 0.74 | 0.41, 1.35 | 0.33 | 0.62 | 0.36, 1.08 | 0.093 |

This is based on complete case dataset. (n=669)

^a^TR = Time Ratio

^b^aTR = adjusted Time Ratio
