# Supplementary material for: Investigating linkage to care between hospitals and primary care clinics for people with TB in rural South Africa
Source: PLoS One. 2023 Aug 14;18(8):e0289830. doi: 10.1371/journal.pone.0289830 (PMC10424851; doi:10.1371/journal.pone.0289830)
Supplement: S1 Checklist — (DOCX) [file pone.0289830.s012.docx]

STROBE Statement—checklist of items that should be included in reports of observational studies

|  | Item No. | Recommendation | Page  No. | Relevant text from manuscript |
| --- | --- | --- | --- | --- |
| **Title and abstract** | 1 | (*a*) Indicate the study’s design with a commonly used term in the title or the abstract | pp. 1 | Investigating linkage to care between hospitals and primary care clinics for people with TB in rural South Africa |
|  |  | (*b*) Provide in the abstract an informative and balanced summary of what was done and what was found | pp. 2 | See abstract. |
| Introduction | | | |  |
| Background/rationale | 2 | Explain the scientific background and rationale for the investigation being reported | pp.3, line 77 | Understanding the magnitude and characteristics of people lost to care in the transfer process can help guide interventions to strengthen the TB care cascade, achieve successful treatment outcomes, and ultimately reduce transmission burden. |
| Objectives | 3 | State specific objectives, including any prespecified hypotheses | pp. 3, line 80 | … to describe the linkage to TB care from hospital to primary care clinic, estimate the proportion of people referred who did not link to care, describe time to linkage-to-care, and identify individual predictors of unsuccessful linkage. |
| Methods | | | |  |
| Study design | 4 | Present key elements of study design early in the paper | pp. 3 | [line 80] This retrospective cohort study …  [line 90] We performed a record review of all patients diagnosed with TB at the 11 study hospitals in Vhembe and Waterberg districts that referred at least two patients to any of the 56 primary care clinics participating in Kharitode TB for TB treatment |
| Setting | 5 | Describe the setting, locations, and relevant dates, including periods of recruitment, exposure, follow-up, and data collection | pp. 3, 4 | [line 87] … in the rural districts of Vhembe and Waterberg, Limpopo province, South Africa. This study was positioned in the six-month “washout” period during which facilities transitioned from contact tracing to facility-based screening (or vice versa).  [line 94] . We then followed a cohort of people diagnosed with drug-sensitive TB at each study hospital from August 1, 2017 to April 30, 2018 via record review for linkage to care. Since we followed each patient to the clinic for linkage after enrolling during this eight-month duration, the follow-up time was variable for different participants.  [line 109] To identify the study population in corresponding hospitals, we used a combination of routine programmatic patient records, including sputum collection logs (at the referring hospital), electronic laboratory records (from the National Health Laboratory Service), and TB hospital and clinic treatment registers (both electronic and paper-based).  Study staff abstracted data from the above sources as well as patient medical files and transfer paperwork at hospitals and clinics. We used a combination of personal identifiers – including name, date of birth, and address – to link patients from hospital to the clinic to which they were referred. After verifying linkage, we abstracted data including age, sex, town or village of residence, date of diagnosis, mode of diagnosis (microbiologically confirmed or clinical), diagnostic test results including date of samples, referring clinic, site of TB (pulmonary or extrapulmonary), retreatment status, HIV status, ART usage at the time of diagnosis, TB symptoms, TB treatment regimen, date of TB treatment initiation, date of referral, and date of linkage to care at local clinic. (See case report form in Supporting table S2) The data were collected and accessed for research between January and July 2018. For each person, we accessed the source data in records only once. |
| Participants | 6 | (*a*) *Cohort study*—Give the eligibility criteria, and the sources and methods of selection of participants. Describe methods of follow-up | pp. 3, 4 | [line 91] We performed a record review of all patients diagnosed with TB at the 11 study hospitals in Vhembe and Waterberg districts that referred at least two patients to any of the 56 primary care clinics participating in Kharitode TB (Supporting table S1) for TB treatment.  [line 101] The study population consisted of all individuals living in the catchment area of one of the 56 study clinics, who were diagnosed with active TB in a hospital and subsequently referred to receive treatment at a Kharitode TB study clinic. We included people of any age diagnosed with pulmonary or extrapulmonary TB using any diagnostic modality, including microbiologically-confirmed, radiologic, or clinical diagnosis. We excluded people with TB documented to be resistant to rifampin.  [line 125] We attempted to reach participants who could not be linked using clinical records by either calling the telephone number found in their records or by visiting their household if telephonic attempts were unsuccessful. We invited such people to participate in a short questionnaire to investigate whether they linked to care anywhere, and the reasons for linking to care at a different clinic or for not linking to care. The interview consisted of up to six questions (see follow-up case report form and participant interview script in Supporting table S3.) |
|  |  | (*b*) *Cohort study*—For matched studies, give matching criteria and number of exposed and unexposed | Not applicable | Not a matched study. |
| Variables | 7 | Clearly define all outcomes, exposures, predictors, potential confounders, and effect modifiers. Give diagnostic criteria, if applicable | pp. 4, 5 | [line 134] … time to linkage as the days elapsed from hospital discharge to initial presentation at the clinic.  [line 156] … association between the outcome and age; sex; HIV status; symptoms of cough, fever, weight loss, and night sweats; category and site of TB; length of hospital admission; and district. |
| Data sources/ measurement | 8* | For each variable of interest, give sources of data and details of methods of assessment (measurement). Describe comparability of assessment methods if there is more than one group | pp. 4 | [line 114] Study staff abstracted data from the above sources as well as patient medical files and transfer paperwork at hospitals and clinics. We used a combination of personal identifiers – including name, date of birth, and address – to link patients from hospital to the clinic to which they were referred. After verifying linkage, we abstracted data including age, sex, town or village of residence, date of diagnosis, mode of diagnosis (microbiologically confirmed or clinical), diagnostic test results including date of samples, referring clinic, site of TB (pulmonary or extrapulmonary), retreatment status, HIV status, ART usage at the time of diagnosis, TB symptoms, TB treatment regimen, date of TB treatment initiation, date of referral, and date of linkage to care at local clinic. |
| Bias | 9 | Describe any efforts to address potential sources of bias | - | None |
| Study size | 10 | Explain how the study size was arrived at | pp. 4 | [line 101] The study population consisted of all individuals living in the catchment area of one of the 56 study clinics, who were diagnosed with active TB in a hospital and subsequently referred to receive treatment at a Kharitode TB study clinic. We included people of any age diagnosed with pulmonary or extrapulmonary TB using any diagnostic modality, including microbiologically-confirmed, radiologic, or clinical diagnosis. We excluded people with TB documented to be resistant to rifampin. |

Continued on next page

| Quantitative variables | 11 | Explain how quantitative variables were handled in the analyses. If applicable, describe which groupings were chosen and why | pp. 5 | [line 156] In all multivariable analyses, we assessed the association between the outcome and age; sex; HIV status; symptoms of cough, fever, weight loss, and night sweats; category and site of TB; length of hospital admission; and district. |
| --- | --- | --- | --- | --- |
| Statistical methods | 12 | (*a*) Describe all statistical methods, including those used to control for confounding | pp. 4, 5 | [line 135] In our primary analysis, we excluded deaths and estimated time to linkage using cause-specific Accelerated Failure Time (AFT) models with robust standard errors to account for clustering at the hospital level.[4] The estimate Time ratio (TR) can be interpreted as percentage change in time to linkage per unit change in exposure variable. We applied multiple imputation with random-sample-observed values to impute time to linkage for patient records with an invalid or missing date of presentation at clinic  []line 146] We censored all observations at 90 days. We used a log-normal distribution for modelling time to linkage because this distribution showed the best fit and had the lowest Akaike Information Criterion (AIC) in comparison with other distributions in the intercept-only model. We further conducted an analysis on a binary outcome defined as linkage to care by 90 days. We used a logistic regression model with random effects for hospital clustering to assess individual characteristics that are associated with linkage. |
|  |  | (*b*) Describe any methods used to examine subgroups and interactions | - | None |
|  |  | (*c*) Explain how missing data were addressed | pp. 5 | [line 139] We applied multiple imputation with random-sample-observed values to impute time to linkage for patient records with an invalid or missing date of presentation at clinic |
|  |  | (*d*) *Cohort study*—If applicable, explain how loss to follow-up was addressed | - | This study examines loss to follow-up during linkage to care. |
|  |  | (*e*) Describe any sensitivity analyses | pp. 5 | [line 140] As a sensitivity analysis, we also estimated time to linkage to care using a competing-risk Accelerated Failure Time (AFT) model with Smooth Rank Regression using Gehan’s weight and Johnson and Strawderman’s sandwich variance estimates to account for the effect of death before linkage as a competing event. |
| Results | | | | |
| Participants | 13* | (a) Report numbers of individuals at each stage of study—eg numbers potentially eligible, examined for eligibility, confirmed eligible, included in the study, completing follow-up, and analysed | pp. 5 | [line 170] The study population included 778 patients who were referred from 11 hospitals in Vhembe and Waterberg districts to the 56 primary care clinics in the study area. (Table 1) Of these, 88 patients (11%) did not continue TB treatment at the primary care clinic to which they were referred, an additional 43 (5.5%) died before linkage to care, and the remaining 647 (83%) linked to care. |
|  |  | (b) Give reasons for non-participation at each stage | - | This study examines loss to follow-up during linkage to care. |
|  |  | (c) Consider use of a flow diagram | Figure 1 | Included |
| Descriptive data | 14* | (a) Give characteristics of study participants (eg demographic, clinical, social) and information on exposures and potential confounders | Table 1 | Included |
|  |  | (b) Indicate number of participants with missing data for each variable of interest | Table 1, Supporting table S4 | Included |
|  |  | (c) *Cohort study*—Summarise follow-up time (eg, average and total amount) | pp. 7, Figure 3 | [line 197] The median time to linkage for the 557 (71.6%) people who linked to care and had a valid date of presentation was 4 days (IQR: 1, 14) |
| Outcome data | 15* | *Cohort study*—Report numbers of outcome events or summary measures over time | pp. 5 | [line 170] … included 778 patients who were referred from 11 hospitals in Vhembe and Waterberg districts to the 56 primary care clinics in the study area. (Table 1) Of these, 88 patients (11%) did not continue TB treatment at the primary care clinic to which they were referred, an additional 43 (5.5%) died before linkage to care, and the remaining 647 (83%) linked to care. |
| Main results | 16 | (*a*) Give unadjusted estimates and, if applicable, confounder-adjusted estimates and their precision (eg, 95% confidence interval). Make clear which confounders were adjusted for and why they were included | Table 2, pp. 7, 8 | [line 197] In the adjusted AFT model, the time to linkage was shorter by half for those reporting cough, compared to those without cough [adjusted Time Ratio (aTR) = 0.53, 95% CI:0.36-0.79, p<0.001]. Similarly, people whose TB was microbiologically confirmed linked to care faster than those diagnosed clinically (aTR = 0.58, 95% CI: 0.34-0.98, p = 0.04). (Table 2) Results were similar when modeling death as a competing risk. (Supporting table S5) No measured characteristic was significantly associated with the risk of death among referred patients. (Supporting table S6) section.  [line 224] People who linked to care within 90 days were younger (adjusted odds ratio comparing ≥50 to <30 years old, aOR = 0.37, 95% CI: 0.18-0.71, p = 0.005) and had higher odds of self-reported cough (aOR = 2.01, 95% CI: 1.26-3.25, p = 0.005), microbiologically confirmed diagnosis (aOR = 1.86, 95% CI: 1.16-3.06, p = 0.012), and residence in Waterberg district (aOR = 1.61, 95% CI: 1.02-2.56, p = 0.041). (Supporting table S7) |
|  |  | (*b*) Report category boundaries when continuous variables were categorized | Table 2 | Included |
|  |  | (*c*) If relevant, consider translating estimates of relative risk into absolute risk for a meaningful time period | - | Different estimates are used: time ratio and odds ratio. |

Continued on next page

| Other analyses | 17 | Report other analyses done—eg analyses of subgroups and interactions, and sensitivity analyses | pp. 8 | [line 228] There was no evidence of statistically significant random variation by hospital. The sensitivity analysis results using complete-case data were consistent with the primary analysis. |
| --- | --- | --- | --- | --- |
| Discussion | | | | |
| Key results | 18 | Summarise key results with reference to study objectives | pp. 9 | [line 232] In this study of 778 people referred out from rural South African hospitals for TB treatment, approximately one in six transferees either died or were lost to follow-up before presenting to the local clinic. People over 50 years old were less likely to link to care, whereas people reporting a cough or with microbiological confirmation were both more likely to link to care and did so faster (in about half the time as their counterparts without a cough or microbiologically confirmed TB |
| Limitations | 19 | Discuss limitations of the study, taking into account sources of potential bias or imprecision. Discuss both direction and magnitude of any potential bias | pp. 10 | [line 267] We used patient records maintained by the health facilities and the national TB program to identify eligible participants and extract information on demographics and TB diagnosis. While valuable in understanding real-world performance, routine data are often not maintained to a high standard of quality. For example, date information was missing or incorrect in 17% of our records. In these cases, we imputed times to linkage based on observed values – but these imputed results could lead to bias in our results. Further, our accounting of deaths is likely an underestimate because some people may have died after linkage to care or interview, and it is likely that some of those for whom we were unable to ascertain linkage or vital status had died. Lastly, socioeconomic characteristics may influence the pathway to care, but the absence of socioeconomic data in routine patient records precluded analysis of this association. |
| Interpretation | 20 | Give a cautious overall interpretation of results considering objectives, limitations, multiplicity of analyses, results from similar studies, and other relevant evidence | pp. 9 | [line 243] Prior estimates of linkage to care at primary care clinics after discharge from hospitals vary widely. Studies in Kwazulu Natal, Western Cape, and Gauteng reported loss to care during linkage of 29%, 36%, and 50%, respectively.[8–10] In comparison, through detailed record review and interview of participants, we were able to confirm linkage to care (or death) in 89% of our rural study population. Our results of better linkage following microbiological diagnosis are consistent with similar findings from the Western Cape.[9] Regarding higher linkage among people with symptomatic cough and microbiological confirmation, these individuals are also more likely to experience more severe symptoms in general, likely increasing the probability of self-directed attempts to seek care at primary care clinics even when they are unaware of, or unable to complete, the initial referral.[11]  Qualitative evidence from Western Cape and Cape Town highlights shortcomings in person-centered care, particularly in the discharge and linkage process.[11–13] People with TB often do not receive adequate resources (e.g., financial support, detailed instructions, contact information) to successfully complete referrals; this is particularly true for people from marginalized communities or with low health literacy. Weak coordination between staff at referring hospitals and local clinics adds to this challenge.[8] Interventions that adopt a person-centered care approach while addressing health system challenges can improve continuity of care.[14] For example, creating an exclusive care and coordination center for people with TB at a tertiary care hospital in Gauteng improved linkage to treatment from 50% to 93%.[15] Similarly, a comprehensive intervention in Western Cape that prepared patients at discharge through education, counselling and coordination with primary care clinics improved linkage from 40% to 92%.[16] |
| Generalisability | 21 | Discuss the generalisability (external validity) of the study results | pp. 9 | [line 247] Our results of better linkage following microbiological diagnosis are consistent with similar findings from the Western Cape.[9] Regarding higher linkage among people with symptomatic cough and microbiological confirmation, these individuals are also more likely to experience more severe symptoms in general, likely increasing the probability of self-directed attempts to seek care at primary care clinics even when they are unaware of, or unable to complete, the initial referral.[11] |
| Other information | |  | | |
| Funding | 22 | Give the source of funding and the role of the funders for the present study and, if applicable, for the original study on which the present article is based | pp. 11 | [line 307] The project through which these data were collected was funded by the National Institutes of Health (R01AI116787). This publication was made possible with help from the Johns Hopkins University Tuberculosis Research Advancement Center, an NIH-funded program (P30AI168436). The content is solely the responsibility of the authors and does not necessarily represent the official views of the NIH. The funders had no role in study design, data collection and analysis, decision to publish, or preparation of the manuscript. |

*Give information separately for cases and controls in case-control studies and, if applicable, for exposed and unexposed groups in cohort and cross-sectional studies.

**Note:** An Explanation and Elaboration article discusses each checklist item and gives methodological background and published examples of transparent reporting. The STROBE checklist is best used in conjunction with this article (freely available on the Web sites of PLoS Medicine at http://www.plosmedicine.org/, Annals of Internal Medicine at http://www.annals.org/, and Epidemiology at http://www.epidem.com/). Information on the STROBE Initiative is available at www.strobe-statement.org.
